# Characteristics of SARS-CoV-2 Omicron BA.5 variants in Shanghai after ending the zero-COVID policy in December 2022: a clinical and genomic analysis

**DOI:** 10.3389/fmicb.2024.1372078

**Published:** 2024-03-28

**Authors:** Pengcheng Liu, Jiehao Cai, He Tian, Jingjing Li, Lijuan Lu, Menghua Xu, Xunhua Zhu, Xiaomin Fu, Xiangshi Wang, Huaqing Zhong, Ran Jia, Yanling Ge, Yanfeng Zhu, Mei Zeng, Jin Xu

**Affiliations:** ^1^Department of Clinical Laboratory, National Children’s Medical Center, Children’s Hospital of Fudan University, Shanghai, China; ^2^Department of Infectious Diseases, National Children’s Medical Center, Children’s Hospital of Fudan University, Shanghai, China; ^3^Shanghai Institute of Infectious Disease and Biosecurity, Fudan University, Shanghai, China

**Keywords:** COVID-19, children, genomics, Omicron BA.5 variants, SARS-CoV-2

## Abstract

**Introduction:**

An unprecedented surge of Omicron infections appeared nationwide in China in December 2022 after the adjustment of the COVID-19 response policy. Here, we report the clinical and genomic characteristics of SARS-CoV-2 infections among children in Shanghai during this outbreak.

**Methods:**

A total of 64 children with symptomatic COVID-19 were enrolled. SARS-CoV-2 whole genome sequences were obtained using next-generation sequencing (NGS) technology. Patient demographics and clinical characteristics were compared between variants. Phylogenetic tree, mutation spectrum, and the impact of unique mutations on SARS-CoV-2 proteins were analysed in silico.

**Results:**

The genomic monitoring revealed that the emerging BA.5.2.48 and BF.7.14 were the dominant variants. The BA.5.2.48 infections were more frequently observed to experience vomiting/diarrhea and less frequently present cough compared to the BF.7.14 infections among patients without comorbidities in the study. The high-frequency unique non-synonymous mutations were present in BA.5.2.48 (N:Q241K) and BF.7.14 (nsp2:V94L, nsp12:L247F, S:C1243F, ORF7a:H47Y) with respect to their parental lineages. Of these mutations, S:C1243F, nsp12:L247F, and ORF7a:H47Y protein were predicted to have a deleterious effect on the protein function. Besides, nsp2:V94L and nsp12:L247F were predicted to destabilize the proteins.

**Discussion:**

Further in vitro to in vivo studies are needed to verify the role of these specific mutations in viral fitness. In addition, continuous genomic monitoring and clinical manifestation assessments of the emerging variants will still be crucial for the effective responses to the ongoing COVID-19 pandemic.

## 1 Introduction

The coronavirus disease 2019 (COVID-19) pandemic caused by severe acute respiratory syndrome coronavirus 2 (SARS-CoV-2) resulted in a global emergence during the past three years since cases were first reported. The emergence of new variants will impose a risk of future surges (Wang et al., 2020; Zhu et al., 2020). Given the widespread and continuous evolution of SARS-CoV-2, numerous variants of concern (VOCs) have emerged and successively dominated multiple waves of the COVID-19 pandemic globally (Boehm et al., 2021). The new VOCs are often associated with increased transmissibility and/or immune evasion properties, which led to their rapid spread globally (Karim and Karim, 2022). Currently, the Omicron variant (B.1.1.529) is the predominant VOC around the world, since its emergence in South Africa in November 2021 (Petersen et al., 2022). From a genomic perspective, it shares several mutations with the previously identified VOCs, such as Alpha (B.1.1.7), Beta (B.1.351), Gamma (P.1), and Delta (B.1.617.2), but it also harbors a large number of specific mutations (Mohapatra et al., 2023). Up to now, a series of Omicron sub-lineages including BA.1 (original Omicron), BA.2, BA.3, BA.4, BA.5, and XBB have emerged and then caused the waves of COVID-19 globally due to further neutralization escape (Ai et al., 2022; Khan et al., 2022; Planas et al., 2022; Qu et al., 2022; Mohapatra et al., 2023). This highlights the importance of continuous genomic monitoring of SARS-CoV-2 variants.

Since the outbreak of COVID-19 in late 2019, China has adhered to policies of zero-COVID for almost three years with strictly enforced lockdowns and other restrictive measures, including social distancing, school closure, mask use, and case isolation (Lai et al., 2020; Liu et al., 2022). Given the attenuated pathogenicity of omicron subvariants and increasing vaccination coverage, China lifted the zero-COVID strategies, notably by announcing the ‘10 measures’ about the optimization of COVID-19 rules on 7 December 2022 (Xinhua, 2022). After that, China experiences a nationwide outbreak of COVID-19. Leung et al. (2023) estimated that the cumulative infection attack rate in Beijing was 75.7% (95% credible interval (CrI): 60.7–84.4) on 22 December 2022 and 92.3% (95% CrI: 91.4–93.1) on 31 January 2023. A recent study by Liang et al. (2023) showed that the cumulative SARS-CoV-2 infection rate rose rapidly to 70% within three weeks after the ending of the zero-COVID policy in Macao. A study conducted in Guangzhou also revealed that the infection attack ratio reached to 80.7% (95% CrI: 72.2–86.8) at 30 days after easing the zero-COVID policy (Huang et al., 2023). Such an unprecedented epidemic raised concerns about specific and real-time data on the viral genetic sequencing, monitoring of variants, and disease impact (World Health Organization, 2022).

Shanghai, with a population of 25 million, is a leading economic center in China. The prevalence of SARS-CoV-2 variants in Shanghai can be considered a snapshot of China. Herein, we report the clinical and genetic characteristics of SARS-CoV-2 infections among children with COVID-19 in Shanghai after ending the zero-COVID policy in December 2022, based on viral genetic sequencing and clinical data.

## 2 Materials and methods

### 2.1 Study population and data collection

This study randomly selected and enrolled 64 pediatric cases with symptomatic COVID-19, who were admitted to the Children’s Hospital of Fudan University in late December 2022. Clinical data were collected via electronic medical charts, including demographic information, clinical symptoms, laboratory findings, and outcomes.

### 2.2 Sample selection and sequencing

Nasopharyngeal swabs obtained from the enrolled cases and confirmed as SARS-COV-2 positive by real-time PCR with cycle threshold (Ct) < 30 were selected for genome sequencing. Viral RNA from the swabs was extracted using an automatic magnetic extraction device and accompanying kit (Daan Gene Co., Ltd) following the manufacturer’s instructions. The SARS-CoV-2 amplicon libraries were generated with a 15 μL viral RNA template, by using the VAHTS RNA Multi-PCR Library Prep Kit according to the manufacturer’s protocol. Libraries were then sequenced on a Nova Seq instrument (Illumina, San Diego, CA, United States) with 2 × 150-bp paired-endreads. Raw reads were trimmed for adapters and filtered for quality (average q20 threshold and read length > 50 nt) using Trimmomatic (version 0.39). The last 8 nucleotides were also removed from all reads. Reference-based assembly was performed with Bowtie2 (version 2.3.5), aligning against the GenBank reference genome MN908947.3. SNPs variants were called through a pipeline based on GATK (version 4.0.6.0), and all SNPs having a minimum supporting read frequency of 50% were retained.

### 2.3 Mutation identification and phylogenetic analysis

Lineages of the SARS-CoV-2 consensus sequences obtained were assigned using Pangolin (version 4.2) (O'Toole et al., 2021). Mutations were identified using Nextclade (version 2.11.0) (Hadfield et al., 2018). All sequences were aligned using the MAFFT (version 7.511) (Katoh et al., 2002) and a Maximum likelihood (ML) phylogenetic tree was reconstructed by IQ-TREE2 (version 2.1.2, COVID-edition) (Minh et al., 2020) using the best-fit model of nucleotide substitution TIM + F + I + I + R2 inferred by ModelFinder (Kalyaanamoorthy et al., 2017) and rooted using Wuhan Hu-1 (MN908947.3) as an outgroup. The phylogenetic tree was visualized and modified with the FigTree (version 1.4.4). The illustrated figures of protein structures with the high-frequency (>50%) unique mutations were prepared using UCSF Chimera (version 1.16) (Pettersen et al., 2004).

### 2.4 Analysis of mutation impact on viral protein function

Preliminary functional analysis of the high-frequency unique non-synonymous mutations in proteins was performed using PredictSNP^1^ (Bendl et al., 2014). Wuhan Hu-1 (MN908947.3) was selected as canonical protein sequence for the analysis. PredictSNP comprises scores from different predictors (MAPP, PhD-SNP, PolyPhen-1, PolyPhen-2, SIFT, SNAP) and uses the information of them to create its own score. PredictSNP then classifies mutations as “neutral” or “deleterious” and transforms the individual confidence scores of each predictor into one comparable scale ranging from 0 to 100%, which represents the percentage of expected accuracy.

### 2.5 Analysis of mutation impact on viral protein stability

The I-Mutant3.0^2^ and DynaMut2^3^ web servers were used for predicting SARS-COV-2 protein conformational stability changes upon the identified high-frequency unique mutations by default pH and temperature. I-Mutant3.0 is a suite of Support Vector Machine (SVM) based predictors and offers the opportunity to predict automatically protein stability changes upon single-site mutations starting from protein sequence alone (Capriotti et al., 2005). DynaMut2 is a structure-based approach for assessing mutation effects on protein stability by using normal mode analysis (NMA) approaches with graph-based distance matrix (Rodrigues et al., 2018). In this study, the wide-type 3D structures of the nucleocapsid (N) protein (PDB ID: 8FD5), spike (S) glycoprotein (PDB ID: 6VXX), nsp2 (PDB ID: 7MSW), nsp12 (PDB ID: 7C2K) and ORF7a protein (PDB ID: 7CI3) were retrieved from Protein Data Bank (PDB). Both tools classify each mutation as stabilizing or destabilizing by providing the predicted Gibbs free energy change (ΔΔG). A positive ΔΔG value corresponds to the mutation predicted to be stabilizing, and a negative value suggests that the mutation can destabilize the protein.

### 2.6 Statistical analysis

Categorical variables were expressed as numbers (%) and compared using Pearson chi-squared or Fisher’s exact tests. Continuous variables were expressed as medians (interquartile range) and compared using Mann–Whitney U tests. All of the tests were two-tailed, and a *p* value <0.05 represented statistical significance. The statistical analyses were conducted in SPSS version 26.0 software (IBM, New York, United States).

## 3 Results

### 3.1 Demographic and clinical characteristics

A total of 64 children with COVID-19 were included in this study. The median age of the patients was 17 months (IQR: 3–43 months), and 64.1% were male. Fever was the most common symptom, observed in 98.4% of the patients, followed by cough (64.1%), shortness of breath (23.4%), and vomiting/diarrhea (21.9%). Abnormal chest imaging was observed in 54.7% of the patients. The median duration of hospitalization was 3 days (IQR: 1.8–4.4 months). Among those, 15 patients (23.4%) had at least one comorbidity. The most common comorbidities were chronic neurological disorders (*n* = 9), followed by chronic haematological disorders (*n* = 4), chronic hepatic disorders (*n* = 2), and solid tumor (*n* = 1). Children with comorbidities were more likely to present shortness of breath, abnormal chest imaging, PICU admission, and longer duration of hospitalization than those without comorbidities (Table 1).

**Table 1:** Clinical characteristics and laboratory findings of children with SARS-CoV-2 infection.

	All patients (*n* = 64)	Without comorbidities (*n* = 49)	With comorbidities (*n* = 15)	*p* value*
Demographics				
Age, median (IQR), m	17 (3–43)	6 (1–34)	96 (28–132)	**<0.001**
Male, *n* (%)	41 (64.1)	31 (63.3)	10 (66.7)	0.058
Symptoms, *n* (%)				
Fever	63 (98.4)	49 (100.0)	14 (93.3)	0.234
Cough	41 (64.1)	32 (65.3)	9 (60.0)	0.708
Shortness of breath	15 (23.4)	7 (14.3)	8 (53.3)	**0.006**
Vomiting/diarrhea	14 (21.9)	12 (24.5)	2 (13.3)	0.577
Wheeze	10 (15.6)	7 (14.3)	3 (20.0)	0.899
Nasal congestion/rhinorrhea	10 (15.6)	10 (20.4)	0 (0.0)	0.134
Hoarseness	5 (7.8)	4 (8.2)	1 (6.7)	1.000
Laboratory findings, median (IQR)				
White blood cells (× 10^9^/L)	6.3 (4.6–8.7)	6.4 (4.9–9.8)	5.5 (3.6–7.7)	0.231
Lymphocytes (× 10^9^/L)	1.8 (1.2–3.5)	2.2 (1.2–4.1)	1.4 (1.1–2.3)	0.063
Neutrophils (× 10^9^/L)	2.6 (1.6–4.5)	2.6 (1.6–4.0)	3.2 (1.5–6.2)	0.496
Hemoglobin (g/L)	121.0 (113.3–128.8)	121.0 (113.5–128.5)	120 (113.0–135.0)	0.668
Platelets (× 10^9^/L)	219.9 (153.0–294.8)	252.0 (158.5–295.5)	195.0 (62.0–235.0)	0.094
Procalcitonin (ng/mL)	0.16 (0.11–0.29)	0.15 (0.11–0.29)	0.19 (0.10–0.50)	0.681
Abnormal chest imaging, *n* (%)	35 (54.7)	23 (46.9)	12 (80.0)	**0.024**
PICU admission, *n* (%)	7 (10.9)	1 (2.0)	6 (40.0)	**<0.001**
Duration of hospitalization, median (IQR), d	3.0 (1.8–4.4)	3.0 (1.5–3.5)	12.0 (3.0–57.0)	**<0.001**

*Comparison between “Without Comorbidities” and “With Comorbidities” groups. Bold indicates significance. IQR, interquartile range; m, months; d, days.

### 3.2 Genotype and phylogenetic analysis

**Figure 1:**
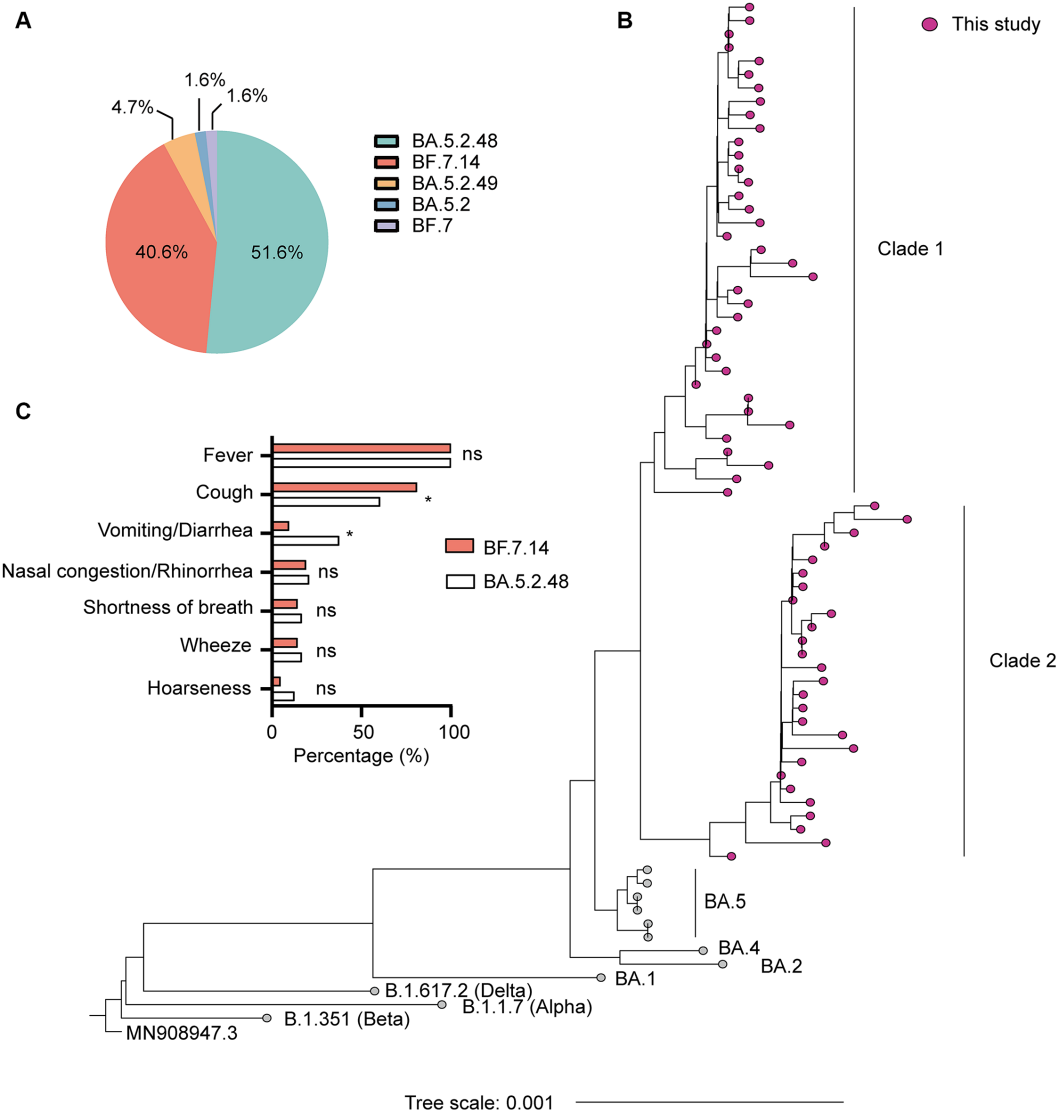
Genetic and clinical characterization of the Omicron variant driving the wave of SARS-CoV-2 outbreak in Shanghai after ending the zero-COVID policy in December 2022. **(A)** The composition of SARS-CoV-2 sub-lineages in this study. BA.5.2.48 and BF.7.14 were the dominant sub-lineages. **(B)** Maximum likelihood (ML) tree of 64 genomes sequenced in this study. The tree was rooted with Wuhan Hu-1 (MN908947.3). **(C)** Comparison of clinical characteristics of infections with BF.7.14 and BA.5.2.48 among children without comorbidities. Statistical evaluations were made with Pearson chi-squared or Fisher’s exact tests. ns, not significant; * significant level at *p* value <0.05.

SARS-CoV-2 genome assemblies were obtained from the enrolled patients. Our genomic monitoring indicated that all cases clustered into the Omicron BA.5.2* lineage, with the dominant lineages of BA.5.2.48 (33/64, 51.6%) and BF.7.14 (26/64, 40.6%). Besides, sporadic detection of other sub-lineages such as BA.5.2.49 (3/64, 4.7%) was also observed (Figure 1A). As expected, the ML tree based on the complete genomes showed that all sequences could be classified into two main clades and clustered tightly with BA.5 lineage (Figure 1B). Thirty-seven sequences belonged to clade 1, including 33 BA.5.2.48, 3 BA.5.2.49, and 1 BA.5.2. The other 27 sequences belonged to clade 2, including 26 BF.7.14 and 1 BF.7 (Figure 1B).

Further, we compared the demographic and clinical characteristics of patients with BA.5.2.48 and BF.7.14 infections. To avoid biases caused by comorbidities, children with and without comorbidities were analyzed individually. The results showed that the BA.5.2.48 infections were more frequently observed to experience vomiting/diarrhea and less frequently present cough compared to the BF.7.14 infections among patients without comorbidities (Table 2) (Figure 1C). Further, to rule out the possibility of other confounding factors, common viruses including norovirus, adenovirus, and rotavirus were detected in the stool samples from the patients with vomiting/diarrhea. The results were all negative for these common viruses. However, there were no significant differences in clinical characteristics and laboratory findings between BA.5.2.48 and BF.7.14 infections among children with comorbidities (Table 3).

**Table 2:** Clinical characteristics and laboratory findings of children without comorbidities according to the infection with different Omicron lineages.

	Total (*n* = 45)	BA.5.2.48 (*n* = 24)	BF.7.14 (*n* = 21)	*p* value*
Demographics				
Age, median (IQR), m	6 (2–34)	9.5 (2–35)	5 (3–24)	0.478
Male, *n* (%)	27 (60.0)	15 (62.5)	12 (57.1)	0.714
Symptoms, *n* (%)				
Fever	45 (100.0)	24 (100.0)	21 (100.0)	1.000
Cough	29 (64.4)	12 (60.4)	17 (81.0)	**0.030**
Vomiting/diarrhea	11 (24.4)	9 (37.5)	2 (9.5)	**0.029**
Nasal congestion/rhinorrhea	9 (20.0)	5 (20.8)	4 (19.0)	1.000
Shortness of breath	7 (15.6)	4 (16.7)	3 (14.3)	1.000
Wheeze	7 (15.6)	4 (16.7)	3 (14.3)	1.000
Hoarseness	4 (8.9)	3 (12.5)	1 (4.8)	0.700
Laboratory findings, median (IQR)				
White blood cells (× 10^9^/L)	6.3 (4.9–8.1)	6.6 (5.9–8.2)	5.4 (4.6–8.1)	0.275
Lymphocytes (× 10^9^/L)	2.2 (1.2–3.8)	2.7 (1.2–4.5)	1.8 (1.1–2.8)	0.453
Neutrophils (× 10^9^/L)	2.6 (1.6–4.0)	2.4 (1.6–3.9)	2.6 (1.8–4.0)	0.502
Hemoglobin (g/L)	121.0 (113.5–127.0)	122.0 (113.3–127.0)	120.0 (112.0–128.5)	0.776
Platelets (× 10^9^/L)	257.0 (168.0–299.0)	202.0 (157.3–293.0)	282.0 (180.0–340.5)	0.280
Procalcitonin (ng/mL)	0.16 (0.12–0.29)	0.15 (0.12–0.29)	0.16 (0.10–0.29)	0.913
Abnormal chest Imaging, *n* (%)	21 (46.7)	10 (41.7)	11 (52.4)	0.517
PICU admission, *n* (%)	1 (2.2)	0 (0.0)	1 (4.8)	0.467
Duration of hospitalization, median (IQR), d	2.5 (1.5–3.5)	2.8 (1.5–3.5)	2.5 (1.5–3.5)	0.917

*Comparison between “BA.5.2.48” and “BF.7.14” groups. Bold indicates significance. IQR, interquartile range; m, months; d, days.

**Table 3:** Clinical characteristics and laboratory findings of children with comorbidities according to the infection with different Omicron lineages.

	Total (*n* = 14)	BA.5.2.48 (*n* = 9)	BF.7.14 (*n* = 5)	*p* value*
Demographics				
Age, median (IQR), m	83 (28–123)	48 (21–144)	120 (62–156)	0.124
Male, n (%)	9 (64.3)	4 (44.4)	5 (100.0)	0.086
Symptoms, n (%)				
Fever	14 (100.0)	9 (100.0)	5 (100.0)	1.000
Cough	9 (64.3)	7 (77.8)	2 (40.0)	0.266
Vomiting/Diarrhea	2 (14.3)	2 (22.2)	0 (0.0)	0.505
Nasal congestion/Rhinorrhea	0 (0.0)	0 (0.0)	0 (0.0)	1.000
Shortness of breath	7 (50.0)	4 (44.4)	3 (60.0)	1.000
Wheeze	3 (21.4)	2 (22.2)	1 (20.0)	1.000
Hoarseness	1 (7.1)	0 (0.0)	1 (20.0)	0.357
Laboratory findings, median (IQR)				
White blood cells (× 10^9^/L)	5.3 (3.4–8.0)	5.2 (3.1–9.6)	5.5 (3.5–8.3)	0.841
Lymphocytes (× 10^9^/L)	1.4 (1.2–2.3)	1.4 (1.0–2.7)	1.3 (1.1–2.3)	0.641
Neutrophils (× 10^9^/L)	3.0 (1.3–6.1)	2.8 (0.5–7.0)	3.6 (1.6–6.4)	0.641
Hemoglobin (g/L)	120.0 (114.5–135.5)	118.0 (104.0–123.5)	135.0 (120.0–140.5)	0.071
Platelets (× 10^9^/L)	210.5 (85.3–243.5)	226.0 (59.5–299.5)	195.0 (137.5–227.0)	0.789
Procalcitonin (ng/mL)	0.19 (0.11–0.59)	0.19 (0.11–0.93)	0.19 (0.09–0.26)	0.537
Abnormal chest imaging, *n* (%)	11 (78.6)	7 (77.8)	4 (80.0)	1.000
PICU admission, *n* (%)	5 (35.7)	3 (33.3)	2 (40.0)	1.000
Duration of hospitalization, median (IQR), d	9 (3–58)	12 (3–45)	5 (3–65)	0.947

*Comparison between “BA.5.2.48” and “BF.7.14” groups. IQR, interquartile range; m, months; d, days.

### 3.3 Mutational analysis

Compared with the reference genome of Wuhan-Hu-1 strain, a total of 194 mutations and deletions were spotted across different genome regions; however, only 111 variant sites with a prevalence of ≥2 sequences were presented. Among the 111 variant sites, there were 70 (63.1%) non-synonymous mutations, 30 (27.0%) synonymous mutations, 5 (4.5%) deletions, and 6 (5.4%) mutations in the untranslated region (UTR) (Table 4). The synonymous mutations were mainly located in non-structural protein (nsp) regions (Table 4).

**Table 4:** Mutations and deletions in 64 sequences of SARS-CoV-2 isolated in this study.

Serial No.	Position	Gene	NT change	Type of mutation	AA change	Sequence count
1	44	5’UTR	C44T	–	–	64
2	210	5’UTR	G210T	–	–	17
3	241	5’UTR	C241T	–	–	58
4	670	nsp1	T670G	Non-synonymous	S135R	64
5	925	nsp2	C925T	Synonymous	–	2
6	1,085	nsp2	G1085T	Non-synonymous	V94L	24
7	1,627	nsp2	C1627T	Synonymous	–	64
8	2,710	nsp2	C2710T	Synonymous	–	29
9	2,790	nsp3	C2790T	Non-synonymous	T24I	64
10	3,037	nsp3	C3037T	Synonymous	–	64
11	4,184	nsp3	G4184A	Non-synonymous	G489S	64
12	4,321	nsp3	C4321T	Synonymous	–	64
13	6,402	nsp3	C6402T	Non-synonymous	P1228L	2
14	7,029	nsp3	C7029T	Non-synonymous	S1437F	2
15	7,528	nsp3	C7528T	Synonymous	–	27
16	8,626	nsp4	C8626T	Synonymous	–	36
17	8,967	nsp4	A8967C	Non-synonymous	K138T	4
18	9,160	nsp4	T9160A	Synonymous		7
19	9,344	nsp4	C9344T	Non-synonymous	L264F	64
20	9,424	nsp4	A9424G	Synonymous	–	64
21	9,534	nsp4	C9534T	Non-synonymous	T327I	64
22	10,029	nsp4	C10029T	Non-synonymous	T492I	64
23	10,198	nsp5	C10198T	Synonymous	–	64
24	10,447	nsp5	G10447A	Synonymous	–	64
25	10,449	nsp5	C10449A	Non-synonymous	P132H	64
26	11,266	nsp6	G11266T	Non-synonymous	L98F	3
27	11,288–11,296	nsp6	deletion	–	S106del, G107del, F108del	64
28	11,365	nsp6	G11365T	Synonymous	–	2
29	11,824	nsp6	C11824T	Synonymous	–	28
30	12,111	nsp8	G12111A	Non-synonymous	S7N	4
31	12,160	nsp8	G12160A	Synonymous	–	63
32	12,310	nsp8	G12310A	Synonymous	–	39
33	12,789	nsp9	C12789T	Non-synonymous	T35I	4
34	12,880	nsp9	C12880T	Synonymous	–	64
35	14,181	nsp12	G14181C	Non-synonymous	L247F	27
36	14,408	nsp12	C14408T	Non-synonymous	P323L	64
37	14,673	nsp12	A14673G	Synonymous	–	3
38	15,026	nsp12	C15026T	Non-synonymous	A529V	2
39	15,714	nsp12	C15714T	Synonymous	–	64
40	16,456	nsp13	T16456C	Non-synonymous	S74P	4
41	16,616	nsp13	C16616A	Non-synonymous	T127N	10
42	16,887	nsp13	C16887T	Synonymous	–	33
43	17,208	nsp13	T17208C	Synonymous	–	33
44	17,410	nsp13	C17410T	Non-synonymous	R392C	64
45	18,087	nsp14	T18087A	Synonymous	–	2
46	18,163	nsp14	A18163G	Non-synonymous	I42V	64
47	19,955	nsp15	C19955T	Non-synonymous	T2163I	64
48	20,055	nsp15	A20055G	Synonymous	–	64
49	20,762	nsp16	C20762T	Non-synonymous	T35I	4
50	21,618	S	C21618T	Non-synonymous	T19I	64
51	21,633–21,641	S	deletion	–	L24del, P25del, P26del, A27S	64
52	21,765–21,770	S	deletion	–	H69del, V70del	64
53	21,809	S	G21809T	Non-synonymous	V83F	2
54	21,987	S	G21987A	Non-synonymous	G142D	64
55	22,200	S	T22200G	Non-synonymous	V213G	36
56	22,578	S	G22578A	Non-synonymous	G339D	64
57	22,599	S	G22599C	Non-synonymous	R346T	25
58	22,674	S	C22674T	Non-synonymous	S371F	63
59	22,679	S	T22679C	Non-synonymous	S373P	63
60	22,686	S	C22686T	Non-synonymous	S375F	63
61	22,688	S	A22688G	Non-synonymous	T376A	63
62	22,775	S	G22775A	Non-synonymous	D405N	64
63	22,786	S	A22786C	Non-synonymous	R408S	64
64	22,813	S	G22813T	Non-synonymous	K417N	64
65	22,882	S	T22882G	Non-synonymous	N440K	64
66	22,917	S	T22917G	Non-synonymous	L452R	64
67	22,992	S	G22992A	Non-synonymous	S477N	64
68	22,995	S	C22995A	Non-synonymous	T478K	64
69	23,013	S	A23013C	Non-synonymous	E484A	64
70	23,018	S	T23018G	Non-synonymous	F486V	64
71	23,055	S	A23055G	Non-synonymous	Q498R	64
72	23,063	S	A23063T	Non-synonymous	N501Y	64
73	23,075	S	T23075C	Non-synonymous	Y505H	64
74	23,403	S	A23403G	Non-synonymous	D614G	64
75	23,525	S	C23525T	Non-synonymous	H655Y	64
76	23,599	S	T23599G	Non-synonymous	N679K	64
77	23,604	S	C23604A	Non-synonymous	P681H	64
78	23,854	S	C23854A	Non-synonymous	N764K	64
79	23,948	S	G23948T	Non-synonymous	D796Y	64
80	24,424	S	A24424T	Non-synonymous	Q954H	64
81	24,469	S	T24469A	Non-synonymous	N969K	64
82	25,000	S	C25000T	Non-synonymous	D1146D	64
83	25,290	S	G25290T	Non-synonymous	C1243F	26
84	25,584	ORF3a	C25584T	Synonymous	–	64
85	25,685	ORF3a	C25685T	Non-synonymous	A98V	2
86	26,060	ORF3a	C26060T	Non-synonymous	T223I	64
87	26,270	E	C26270T	Non-synonymous	T9I	64
88	26,408	E	C26408T	Non-synonymous	S55F	4
89	26,529	M	G26529A	Non-synonymous	D3N	63
90	26,577	M	C26577G	Non-synonymous	Q19E	64
91	26,709	M	G26709A	Non-synonymous	A63T	64
92	27,012	M	C27012T	Synonymous	–	38
93	27,038	M	A27038G	Synonymous	–	6
94	27,513	ORF7a	C27513T	Synonymous	–	38
95	27,532	ORF7a	C27532T	Non-synonymous	H47Y	26
96	27,807	ORF7b	C27807T	Synonymous	–	64
97	27,889	UTR	C27889T	–	–	64
98	28,271	UTR	A28271T	–	–	64
99	28,311	N	C28311T	Non-synonymous	P13L	64
100	28,330	N	A28330G	Synonymous	–	64
101	28,361	N	G28361T	Non-synonymous	S33F	23
102	28,362–28,370	N	deletion	–	E31del, R32del, S33del	64
103	28,371	N	G28371T	Non-synonymous	S33F	23
104	28,792	N	A28792T	Synonymous	–	11
105	28,881–28,882	N	G28881A, G28882A	Non-synonymous	R203K	44
106	28,881–28,882	N	G28881C, G28882A	Non-synonymous	R203T	9
107	28,883	N	G28883C	Non-synonymous	G204R	57
108	28,994	N	C28994A	Non-synonymous	Q241K	19
109	29,510	N	A29510C	Non-synonymous	S413R	64
110	29,632	ORF10	C29632T	Synonymous	–	27
111	29,734–29,759	3’UTR	Deletion	–	–	64

Only variant sites with a prevalence of ≥ 2 sequences were presented in this table. NT, nucleotide; AA, amino acid.

**Figure 2:**
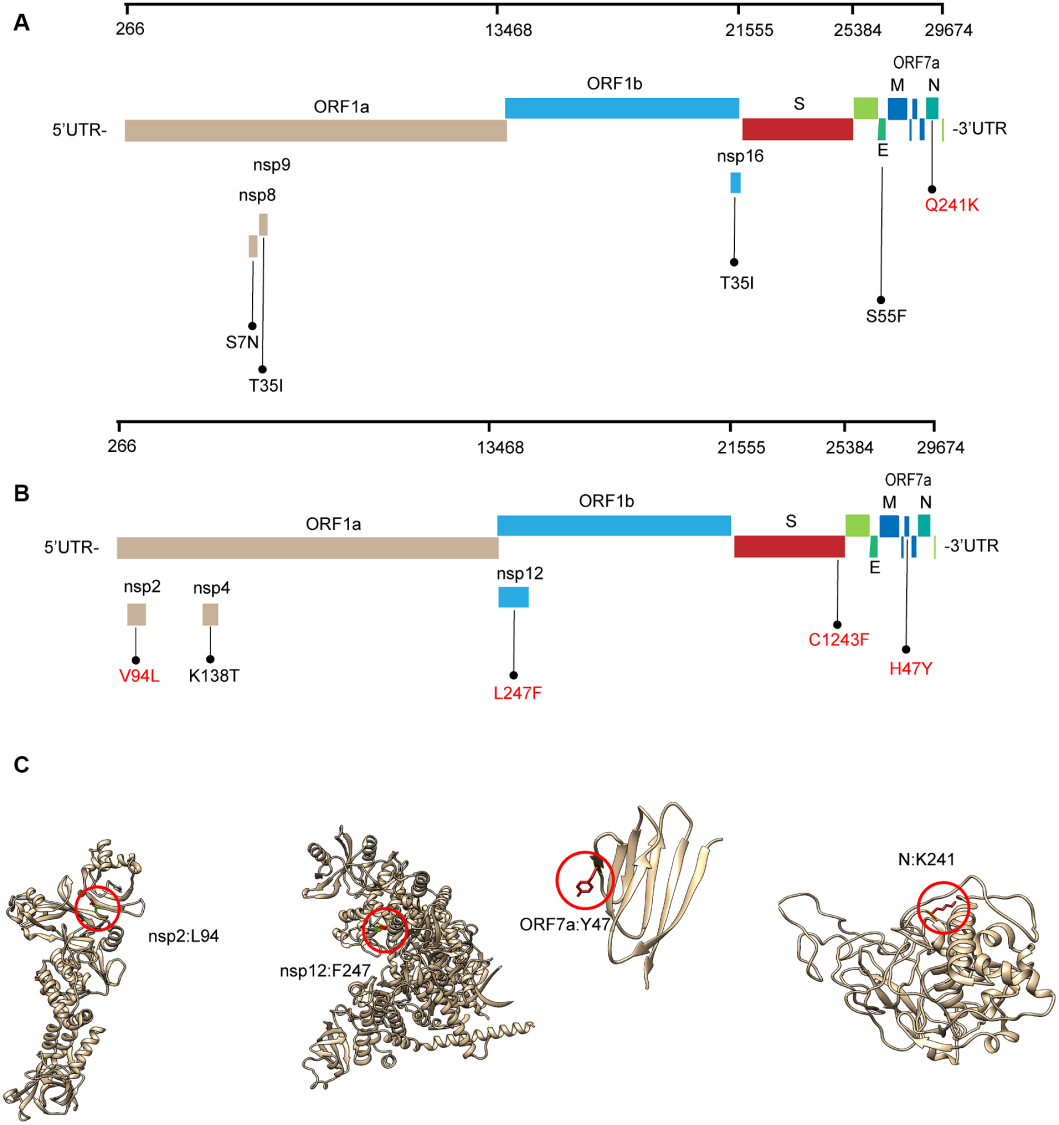
Schematic representation of the unique amino acid mutations of the variants in this study. The schematic diagram of BA.5.2.48 **(A)** and BF.7.14 **(B)** unique amino acid mutation sites on the SARS-CoV-2 genome. The high frequency unique mutations are shown in red. **(C)** The location of the high-frequency unique mutations in the protein crystal structures. V94L mutation is located in the nsp2 N-terminal. L247F mutation is located in the NiRAN domain, which lies at the N terminal end of the RdRp (nsp12) domain. H47Y mutation is located in the ectodomain of the ORF7a protein. Q241K mutation is located in an intrinsically disordered region of the N protein, which connects the N-terminal domain and the C-terminal domain. Mutation positions are framed in red circles, and mutant residues are represented in stick form. C1243F mutation is located at the cytoplasmic region of the spike protein without a resolution crystal structure.

Most of these variant sites were in common with the known mutations in their parental lineages (BA.5.2 for BA.5.2.48, and BF.7 for BF.7.14). However, we found 5 unique non-synonymous mutations in BA.5.2.48 and BF.7.14, respectively, with high frequency (>50%) (Table 5). Among these unique mutations, N:Q241K is a characteristic mutation of BA.5.2.48 linage and nsp2:V94L, nsp12:L247F, S:C1243F, and ORF7a:H47Y are the characteristic mutations of BF.7.14 linage. Figure 2 is a graphical representation that shows the location of these unique mutations in each region of the complete SARS-CoV-2 genome and the high frequency unique mutations in the protein crystal structures.

**Table 5:** Unique non-synonymous mutations in BA.5.2.48 and BF.7.14 compared with their parental lineages.

Lineage	Genomic region	Nucleotide mutation	AA mutation	Frequency
BA.5.2.48	nsp8 (ORF1ab)	G12111A	S7N	4/33 (12.1%)
	nsp9 (ORF1ab)	C12789T	T35I	4/33 (12.1%)
	nsp16 (ORF1ab)	C20762T	T35I	4/33 (12.1%)
	E	C26408T	S55F	4/33 (12.1%)
	N	C28994A	**Q241K**	19/33 (57.6%)
BF.7.14	nsp2 (ORF1ab)	G1085T	**V94L**	23/26 (88.5%)
	nsp4 (ORF1ab)	A8967C	K138T	3/26 (11.5%)
	nsp12 (ORF1ab)	G14181C	**L247F**	26/26 (100.0%)
	Spike	G25290T	**C1243F**	26/26 (100.0%)
	ORF7a	C27532T	**H47Y**	26/26 (100.0%)

Only mutation frequency more than 10% are considered. High-frequency (>50%) mutations are highlighted in bold. N:Q241K is a characteristic mutation of BA.5.2.48 linage. Nsp2:V94L, nsp12:L247F, S:C1243F, and ORF7a:H47Y are the characteristic mutations of BF.7.14 linage. BA.5.2 (GISAID accession ID: EPI_ISL_16614598) was used as the parental lineage for the BA.5.2.48; BF.7 (GISAID accession ID: EPI_ISL_16327362) was used as the parental lineage for the BF.7.14. AA, amino acid.

**Figure 3:**
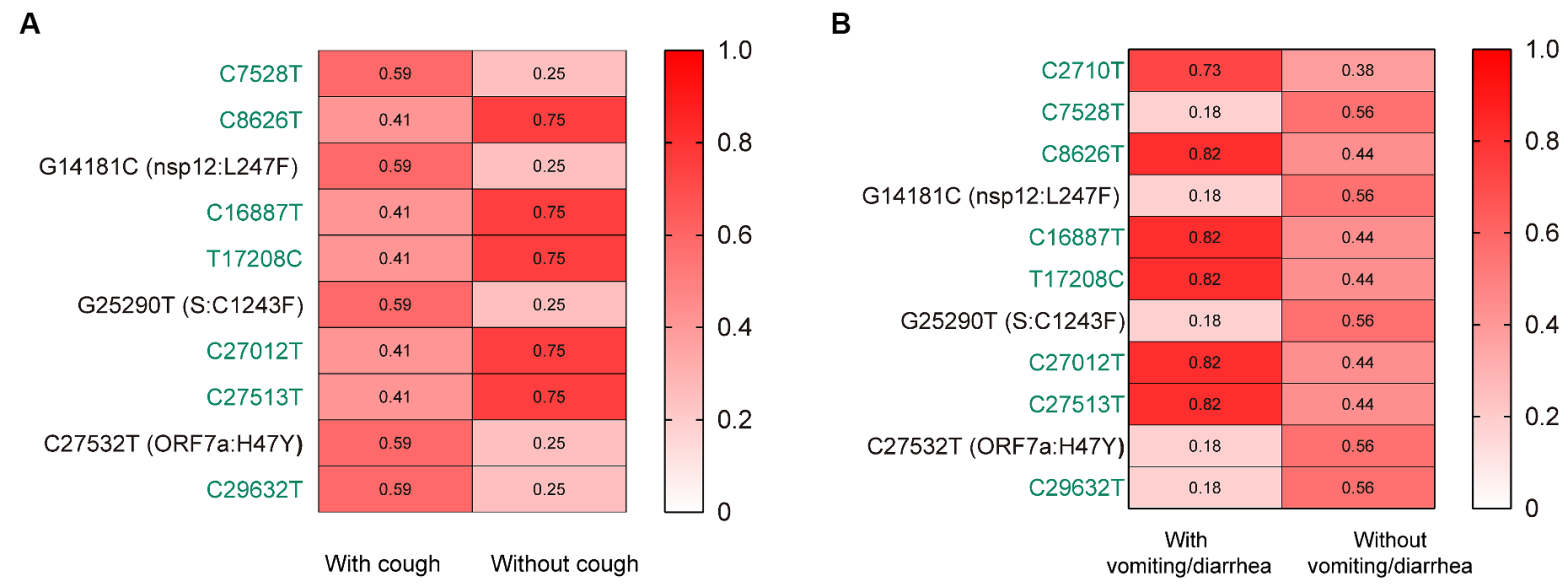
The associations of the mutations with the symptoms of cough **(A)** and vomiting/diarrhea **(B)**. The mutation frequencies between groups were compared using Pearson chi-squared or Fisher’s exact tests. Only mutations with statistically significant difference in frequencies between groups are presented. Only children without comorbidities were included. Color gradient indicates mutation frequencies. Synonymous mutations are colored in green.

Further, we assessed the associations of the mutations with the symptoms of vomiting/diarrhea and cough. Interestingly, we found that the frequency of nsp12:L247F, S:C1243F, and ORF7a:H47Y (characteristic mutations of BF.7.14) was significantly higher among those with cough than without (Figure 3A). Besides, the frequency of these three mutations was lower among those with vomiting/diarrhea than without (Figure 3B).

### 3.4 Effect of mutations on protein function

**Figure 4:**
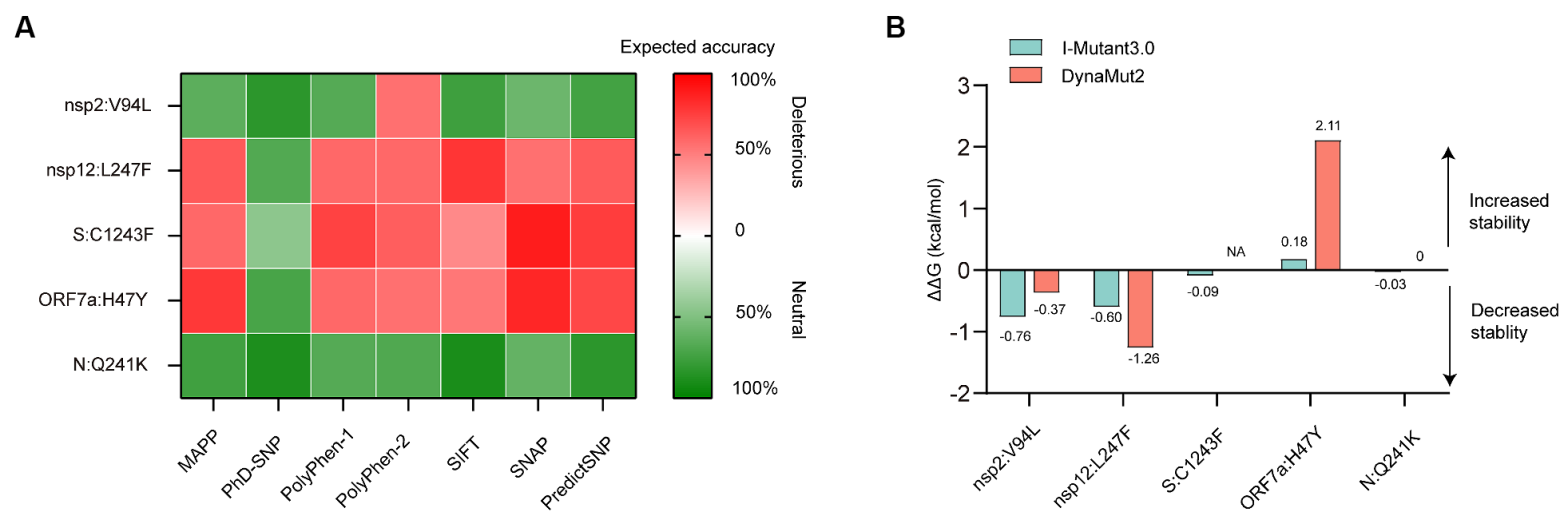
*In silico* analysis of protein function and structural stability changes upon the high-frequency unique mutations using different tools. **(A)** Effect of the mutations on protein function. The red color stands for deleterious mutation, whereas the green color represents neutral mutation. **(B)** Effect of the mutations on protein structural stability. Site 1,243 without resolution in the spike (S) protein crystallographic structure, located at the cytoplasmic region was not analyzed for energy estimation by the structure-based tool Dynamut2. NA, not available.

The predicted effects of pathogenicity for the high-frequency mutations are show in Figure 4A. A mutation was classified as deleterious only when it was predicted as deleterious by more than three tools. Our results revealed that the Q241K mutation in N protein and the V94L mutation in nsp2 were predicted to have a neutral effect on the protein function. However, L247F mutation in nsp12, C1243F mutation in S protein, and H47Y mutation in ORF7a protein were predicted to be deleterious by the consensus classifier.

### 3.5 Effect of mutations on protein stability

Structural stability of proteins due to the high-frequency mutations were analyzed using I-Mutant and DynaMut stability predictors. The tools provided almost consensus predicted results. The V94L mutation in nsp2, as well as the L247F mutation in nsp12, destabilized the proteins. While the H47Y mutation increased the stability of structure of the ORF7a protein. The Q241K mutation in the N protein and C1243F mutation in the S protein had little effect on the protein stability due to the low absolute ΔΔG values (Figure 4B).

## 4 Discussion

In this study, we have reported the first comprehensive clinical and viral genomic analysis of SARS-CoV-2 infections among children hospitalized with COVID-19 in Shanghai after ending the zero-COVID policy. Whole genome sequencing of samples obtained from the enrolled 64 pediatric patients revealed that all cases clustered into the Omicron BA.5.2* lineage, with the dominant omicron sub-lineages of BA.5.2.48 and BF.7.14. This result was in line with our previous study based on 8,254 SARS-CoV-2 complete genomes available on the GISAID database from the Chinese mainland during December 2022 and January 2023, which indicated that the genomes corresponded to 88 Pango-nomenclature-system-named subvariants, with the dominant lineages of BA.5.2.48 (4,881/8254, 59.1%) and BF.7.14 (2,223/8254, 26.9%), and the proportion of these dominant lineages were not significant changed over the two months of outbreak (Liu and Xu, 2023). In addition, BA.5.2.48 and BF.7.14 were also found to be the dominant lineages among SARS-CoV-2 positive passengers on flights from China to Italy in late December 2022 (Novazzi et al., 2023). However, the lineages found to be dominant internationally during the same period, such as BQ.1, BQ.1.1, and XBB.1.5 were quickly cleared and did not prevail in China (Liu and Xu, 2023). Taken together, all these results demonstrated that the emerging BA.5.2.48 and BF.7.14 were the absolutely dominant drivers of the current COVID-19 outbreak after ending the zero-COVID policy, which could be attributed to the high fitness of lineages or a random founder effect in China.

Fever and cough were the most common symptoms among children with COVID-19 in this study. This result was consistent with the earlier community outbreak in Shanghai driven by BA.2.2.1 sub-lineage in spring 2022 (Ao et al., 2022; Ling et al., 2022; Shen et al., 2023). However, the demographic characteristics, clinical symptoms, laboratory findings, and outcomes might vary between different SARS-CoV-2 variants in children hospitalized with COVID-19 (Boncuoglu et al., 2022; Quintero et al., 2022; Tagarro et al., 2022; Sahin et al., 2023). Consequently, we further compared the clinical features of children with BA.5.2.48 and BF.7.14 infections. We found that the BA5.2.48 infections were more frequently observed to experience vomiting/diarrhea and less frequently present cough compared to the BF.7.14 infections among patients without comorbidities. To figure out susceptible mutations related to the variation of symptoms, we assessed the associations of the mutations with the symptoms of vomiting/diarrhea and cough. We found that the frequency of the characteristic mutation nsp12:L247F, S:C1243F, and ORF7a:H47Y was significantly varied among those with cough or vomiting/diarrhea than without. These observations suggest that these three characteristic mutations might contribute to the variation of symptoms between children infected with BF.7.14 and BA.5.2.48 by affecting the tissue tropism of the variants or other mechanisms. However, possibly arose bias due to the limited sample size in this study. Besides, the results might also be affected by the demographic characteristics, especially age. Thus, the interpretation of the results must be cautious and might not precisely reflect the broader population. Given the rapid evolution of SARS-CoV-2, further studies are still needed to elucidate the clinical features and severity of different variants with specific mutations.

Adaptive mutations in the SARS-CoV-2 genome could alter its pathogenic potential, and at the same time would increase the infectivity and immune escape capacity. Single amino acid changes are worth monitoring because they can be phenotypically relevant. Perhaps one of the best exemplars of the impacts of amino acid changes in the SARS-CoV-2 is the D614G mutation in S protein. D614G substitution was first identified in early 2020 and rapidly spread throughout the global population by increasing the infectivity and stability of virion (Korber et al., 2020; Zhang et al., 2020; Plante et al., 2021). With this background, we further investigated the unique amino acid changes in the BA.5.2.48 and BF.7.14 sub-lineages, and predicted the effects of these mutations on the stability and function of viral proteins. Stability is a parameter which is crucial to judge the functional and structural activity of a protein. Protein stability dictates the conformational structure of the protein, thereby determining its function. Any change in protein stability may cause misfolding, degradation or aberrant conglomeration of proteins. Understanding the stability changes in SARS-CoV-2 proteins is essential for predicting virus infectivity. Changes in Gibbs free energy of unfolding (ΔΔG) between the wild-type and mutant proteins could predict the effects of mutations on the stability of protein structure (Pan et al., 2022). In order to ascertain the significance of mutations on protein function, we analyzed the pathogenicity of the mutations as deleterious or neutral. It is important to note that in case of protein, damaging mostly defines instability. Generally, this is used for human proteins. As a consequence, if the human protein is damaging in nature because of mutations, then the human protein–protein interactions may occur with high or low binding affinity. Now in case of virus, similar consequences may happen, which means if the virus protein is damaged because of mutations, it may interact with human proteins with similar binding affinity. As a result, the virus may acquire characteristics like transmissibility, escaping antibodies. For example, the D614G was predicted to be deleterious and instable using I-mutant and PredictSNP servers. Thus, the basic premise for the study was that mutations will be contributing to the viral evolution only if they are deleterious and neutral mutations would not be affecting the protein function (Laskar and Ali, 2021).

We found 5 high-frequency amino acid changes (N: Q241K, nsp2: V94L, nsp12: L247F, S: C1243F, ORF7a: H47Y) in the BA.5.2.48 and BF.7.14 sub-lineages. Among these mutations, the C1243F mutation in S protein, L247F mutation in nsp12, and H47Y mutation in ORF7a protein were predicted to have a deleterious effect on the protein function. S protein decorates the surface of coronavirus and plays a critical role in viral entry (Gallagher and Buchmeier, 2001). It comprises two functional subunits responsible for binding to the host cell receptor (S1 subunit) and membrane fusion (S2 subunit) (Walls et al., 2020). In the S1 subunit, there is an N-terminal domain (14–305 residues) and a receptor-binding domain (RBD, 319–541 residues); the fusion peptide (FP) (788–806 residues), heptapeptide repeat sequence 1 (HR1) (912–984 residues), HR2 (1,163–1,213 residues), transmembrane domain (1,213–1,237 residues), and intracellular domain (1,237–1,273 residues) comprise the S2 subunit (Huang et al., 2020). The C1243F mutation is located in the intracellular domain of S2 subunit. The mutations in the intracellular domain are unlikely to drive immune evasion. However, the mutations in this domain may affect the S protein expression at the cell surface and syncytia formation by mediating intracellular trafficking and membrane location of S protein (Cattin-Ortola et al., 2021; Li et al., 2022).

nsp12, also named RNA-dependent RNA polymerase (RdRp), catalyzes the synthesis of viral RNA and thus plays a central role in the replication and transcription cycle, with the assistance of nsp7 and nsp8 as cofactors. The structure of the nsp12 contains a right-hand RdRp domain (367–920 residues) and a nidovirus RdRp-associated nucleotidyltransferase domain (NiRAN, 60–249 residues) (Gao et al., 2020). The L247F mutation is located in the NiRAN domain, which lies at the N terminal end of the RdRp. Although the NiRAN domain is essential for viral propagation, its functions during the viral life cycle remain unclear. A recent study revealed that the NiRAN domain catalyzes the covalent link of RNA 5′ end to the first residue of nsp9, thus being an intermediate to form cap core (GpppA) with GTP catalyzed again by NiRAN (Yan et al., 2022). Therefore, the L247F mutation in nsp12 may affect the SARS-CoV-2 replication in the host cells.

ORF7a protein is a type-I transmembrane protein, consisting of an N-terminal signaling region (1–15 residues), an immunoglobulin-like ectodomain (16–96 residues), a hydrophobic transmembrane domain (97–116 residues), and a typical endoplasmic reticulum retention motif (117–121 residues) (Zhou et al., 2021). The H47Y mutation is located in the ectodomain. A recent study suggested that the Immunoglobulin-like fold ectodomain of the ORF7a interacts with high efficiency to the CD14+ monocytes in human peripheral blood, and ORF7a may also suppress the antigen-presenting ability of these monocytes and trigger the significant upregulation of multiple proinflammatory cytokines (Zhou et al., 2021). Further *in vitro* and *in vivo* studies are needed to verify the role of ORF7a: H47Y mutation in viral fitness.

## 5 Conclusion

Our results revealed that the current large-scale COVID-19 outbreak in Shanghai after ending the zero-COVID policy was driven by the emerging BA.5.2.48 and BF.7.14 variants with unique deleterious mutations. In addition, this study described the clinical characteristics of pediatric cases infected with BA.5.2.48 and BF.7.14. Continuous genomic monitoring and clinical manifestation assessments of the emerging variants will be crucial for countering the ongoing COVID-19 pandemic.

## Data availability statement

The datasets presented in this study can be found in online repositories. The names of the repository/repositories and accession number(s) can be found at: https://gisaid.org, EPI_ISL_17371203 to EPI_ISL_17371266.

## Ethics statement

The studies involving humans were approved by Children’s Hospital of Fudan University. The studies were conducted in accordance with the local legislation and institutional requirements. Written informed consent for participation was not required from the participants or the participants’ legal guardians/next of kin in accordance with the national legislation and institutional requirements.

## Author contributions

PL: Conceptualization, Formal analysis, Methodology, Writing – original draft, Writing – review & editing. JC: Conceptualization, Investigation, Resources, Writing – review & editing. HT: Data curation, Investigation, Writing – review & editing. JL: Investigation, Validation, Writing – review & editing. LL: Resources, Validation, Writing – review & editing. MX: Investigation, Resources, Writing – review & editing. XZ: Investigation, Resources, Writing – review & editing. XF: Investigation, Resources, Writing – review & editing. XW: Methodology, Resources, Writing – review & editing. HZ: Investigation, Resources, Writing – review & editing. RJ: Investigation, Resources, Writing – review & editing. YG: Investigation, Resources, Writing – review & editing. YZ: Investigation, Resources, Writing – review & editing. MZ: Conceptualization, Project administration, Supervision, Writing – review & editing. JX: Conceptualization, Project administration, Supervision, Writing – review & editing.

## Footnotes

^1^ https://loschmidt.chemi.muni.cz/predictsnp

^2^ http://gpcr2.biocomp.unibo.it/cgi/predictors/I-Mutant3.0/I-Mutant3.0.cgi

^3^ http://biosig.unimelb.edu.au/dynamut
